# Hypothyroidism predicts worsened prognosis in patients undergoing percutaneous coronary intervention

**DOI:** 10.3389/fcvm.2022.984952

**Published:** 2022-11-29

**Authors:** Ben Cohen, Tamir Bental, Liat Perl, Hana Vaknin Assa, Pablo Codner, Katia Orvin, Yeela Talmor Barkan, Amos Levi, Ran Kornowski, Leor Perl

**Affiliations:** ^1^Department of Cardiology, Rabin Medical Center, Petah Tikva, Israel; ^2^Faculty of Medicine, Tel Aviv University, Tel Aviv, Israel; ^3^Pediatric Endocrinology and Diabetes Unit, Dana-Dwek Children’s Hospital, Tel Aviv Sourasky Medical Center, Tel Aviv, Israel

**Keywords:** hypothyroid, percutaneous coronary intervention (PCI), major cardiovascular adverse event, outcomes, ischemic heart disease

## Abstract

**Background:**

The link between thyroid dysfunction and cardiovascular disease is well established. Hypothyroidism has been significantly associated with increased risk of dyslipidemia, atherosclerosis and heart failure. However, little is known regarding its effect on patients undergoing percutaneous coronary intervention (PCI).

**Aim:**

The aim of study was to examine the impact of concomitant hypothyroidism on mortality and major adverse cardiac event (MACE) in patients undergoing PCI.

**Methods:**

The Rabin Medical Center PCI registry includes all consecutive patients who have undergone PCI between 2004 and 2020. We identified patients with prior diagnosis of hypothyroidism, and compared rates of mortality and MACE (comprising death, myocardial infarction, target vessel revascularization and/or coronary bypass surgery).

**Results:**

Among 28,274 patients, 1,922 (6.8%) were found to have hypothryoidism. These patients were older (70.3 ± 10.4 vs. 66.0 ± 11.8 y.o, *P* < 0.001) and more likely to be women (34.2% vs. 26.1%, *P* < 0.001). They had a higher prevalence of atrial fibrillation (10.8% vs. 7.7%, *P* < 0.001), chronic renal dysfunction (25.1% vs. 18.7%, *P* = 0.04) and dementia (2.9% vs. 1.8%, *P* = 0.004). PCI was performed on ACS setting in 52–54% of patients in both groups (*p* = 0.569). Unadjusted 5-year rates of all-cause mortality (26.9% vs. 20.3%, *P* < 0.001) and MACE (40.3% vs. 29.4%, *P* < 0.001) were higher for hypothyroid patients. A propensity match score was able to form 672 matched pairs of HT and control patients, showing similar results. Moreover, following multivariate analysis, TSH as a continuous parameter was associated with a higher risk of mortality and MACE (HR, 1.06 per additional 1 mIU/L; CI, 1.02–1.11; *P* < 0.001 and HR, 1.07; CI, 1.02–1.12; *P* < 0.001, respectively) at 5-year follow up.

**Conclusion:**

In our study, hypothyroidism confers worse outcomes in patients undergoing PCI. Further research is needed to establish effective ways to mitigate this augmented risk.

## Introduction

Thyroid hormones are known to affect the cardiovascular system. Both hypo- and hyperthyroidism have been linked to cardiovascular disease (1, 2). Hypothyroidism (HT) has been suggested as a causative factor in dyslipidemia and metabolic syndrome (3). Hyperthyroidism, on the other hand has been associated with high-output heart failure, dilated cardiomyopathy and atrial fibrillation (4). That is why determination of the thyroid hormonal status is indicated in many cardiovascular diseases, as reflected in the European Society of Cardiology 2021 heart failure and the European Society of Cardiology 2020 atrial fibrillation guidelines (5, 6).

The prevalence of HT is between 4 and 10% of the population (7). HT is associated with cardiovascular changes of arterial compliance, diastolic blood pressure, endothelial dysfunction and hyperlipidemia (8). Hence, it is not surprising that HT and to a lesser degree subclinical hypothyroidism (SHT) are associated with an accelerated atherosclerosis and a higher prevalence of ischemic heart disease (9, 10). The association of HT with atherosclerosis seems to be mediated with intima-media thickness and the above mentioned pathophysiological entities. However, the exact mechanisms in which it may hasten atherosclerosis are not fully understood (11).

Whether thyroid hormone status matters in patients with established ischemic heart disease, in terms of mortality, re-infarction, restenosis and in stent thrombosis has long been a subject of debate. Previous studies imply HT, and to a lesser extent- SHT, are associated with a higher incidence of major adverse cardiovascular or cerebrovascular events (MACCE) compared with euthyroid patients undergoing percutaneous coronary intervention (PCI) (12–15). However, as previous studies were based on small groups of patients, and usually defined thyroid hormonal status based on a single measurement done in the setting of acute coronary syndrome, we thought that further research is needed.

**FIGURE 1:**
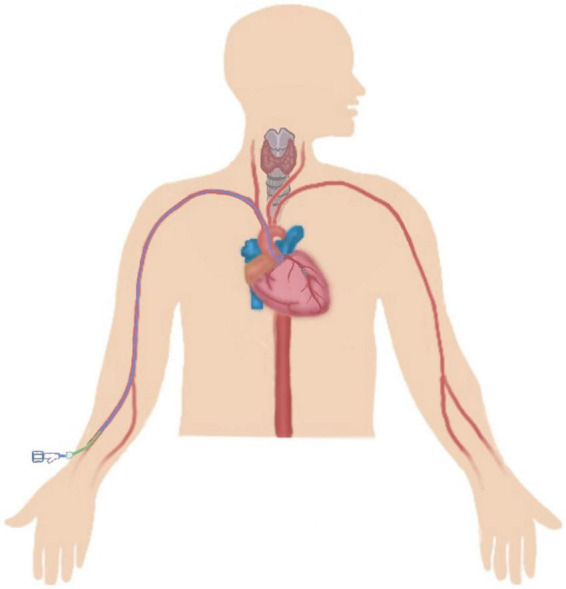
Central illustration.

Understanding the additional relative risk posed in patients with HT, and whether it is preventable with thyroid replacement therapy, may impact a substantial percentage of patients undergoing PCI. The following study attempts to shed light on the correlation between HT and prognosis in patients with concomitant ischemic heart disease undergoing PCI (Figure 1).

## Materials and methods

### Patients and setting

The current study is an observational study based on the prospective Rabin Medical Center PCI registry. This database includes all consecutive patients who have undergone PCI at the Rabin Medical Center, Israel, between 2004 and 2020 PCI at two hospitals: the Beilinson and HaSharon medical centers. We identified patients with prior diagnosis of HT and compared their baseline and procedural characteristics and long terms prognosis to euthyroid patients. Data before and after hospitalization were obtained from the hospital’s and the Clalit healthcare services’ (largest healthcare provider in Israel) databases. Mortality data were obtained from the national Ministry of Interior database.

The obtained data included demographic and clinical characteristics, cardiovascular risk factors, co-morbidities (based on ICD-9 diagnoses codes), procedural characteristics, and clinical workup (e.g., blood tests, echocardiography) as was previously reported (16). Most patients who underwent PCI, regardless of etiology, were hospitalized for at least 1 day thereafter. In addition to detailed information on the medical conditions and medications of all patients, we extracted thyroid stimulating hormone (TSH) values 6 months before PCI and considered values above 10.0 mIU/L as an indication of undiagnosed HT and values above 5 mIU/L in the presence of HT as a suboptimal treatment. Patients with hyperthyroidism or SHT were excluded from the study, as well as those with missing data regarding TSH levels, baseline characteristics or outcomes. Included in the final study group were all patients with an established diagnosis of HT or permanent thyroid replacement therapy according to their electronic medical records. They were compared to patients with no known diagnosis of HT and TSH levels within the normal range. Patients were not screened for development of thyroidal illness post PCI unless diagnosis was made as part of the hospital admission in which the PCI was performed.

The hospital’s ethics committee approved the study, which was performed in accordance with the Helsinki declaration.

### Interventional procedure

All patients provided an explicit written informed consent before undergoing cardiac catheterization and PCI as indicated. Administration of anticoagulants before and after PCI was in accordance with the clinical setting (acute versus chronic coronary syndrome) and the latest guidelines which have been updated throughout the years of this study.

All patients were treated with aspirin 200–300 mg before PCI, clopidogrel 300–600 mg, prasugrel 60 mg or ticagrelor 180 mg (in acute coronary syndromes) either before PCI (pretreatment, in cases of acute coronary syndromes) or immediately after completion of the procedure (in elective cases). Glycoprotein IIb/IIIa inhibitors were used during the procedure and immediately following the PCI, at the discretion of the operator. The choice of the type of coronary stent and other adjunct therapy were left to the discretion of the primary operator and shifted exclusively toward drug eluting stents and *trans*-radial access in recent years. All stents were implanted with moderate-to-high deployment pressure (∼12 atm) with or without post dilatation as needed. All patients received recommendation to continue dual antiplatelet therapy with aspirin 100 mg daily and a P2Y12 inhibitor (clopidogrel, prasugrel, or ticagrelor) for at least 12 months after the PCI.

### Study end points

Immediate and in-hospital clinical events were prospectively documented in the Rabin Medical Center PCI registry. Information on patient outcomes (mortality and other clinical outcomes) was drawn from the databases listed above on December 2021.

Outcomes included all-cause mortality and major adverse cardiac event (MACE), which comprised death, myocardial infarction (MI) excluding peri-procedural MI, need for target vessel revascularization, and/or coronary artery bypass surgery (CABG) at 1 and 5 years.

### Statistical analysis

Continuous data are presented as median and interquartile range, and categorical data, as frequency (%). Student *t*-test or analysis of variance was used to compare continuous variables between groups, and Chi-square was used for categorical variables. The normality of variable distributions was assessed using the Kolmogorov–Smirnov test. Survival curves were constructed using the Kaplan–Meier method and compared using log-rank test. Cox regression analysis was performed to determine independent predictors of the primary end points, assessing the contribution of increased TSH as a continuous parameter and known baseline cardiovascular risk factors. Finally, due to several differences in baseline characteristics, we compiled a cohort of propensity score matched patients with a 1:1 ratio between patients with HT and controls. The propensity score was derived from a multivariate logistic regression model that included the presence of hypothyroidism, considered as the independent (outcome) variable, and all baseline clinical characteristics and procedural characteristics as covariates. The propensity score matched cohort was analyzed for the main combined outcome. All statistical analyses were performed with IBM SPSS version 28.0 (IBM Corp. Armonk, NY, United States). A *P*-value of < 0.05 was considered statistically significant.

## Results

**FIGURE 2:**
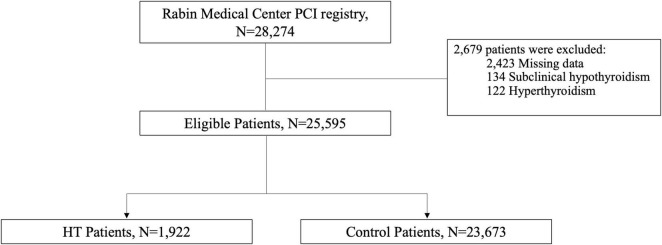
Description of the study cohort.

We have analyzed information based on 28,274 patients who have undergone PCI between the years 2004 to 2020. After exclusion of 2,679 patients due to missing data regarding thyroid function, evidence of hyperthyroidism or SHT, 25,595 patients were eligible for our final analysis. 1,922 of these patients, 6.8% of the total PCI cohort, were diagnosed as HT based on the above criteria (Figure 2).

Patients with HT were ∼4 years older (70.4 ± 10.4 vs. 66.0 ± 11.8 y.o, *P* < 0.001), were more likely to be women (34.2% vs. 26.1%, *P* < 0.001). They had a higher prevalence of atrial fibrillation (10.8% vs. 7.7%, *P* < 0.001), chronic kidney disease (251% vs. 18.7%, *P* = 0.04), anemia (54.1% vs. 50.8%, *P* = 0.052), and demetia (2.9% vs. 1.8%, *P* = 0.004). Moreover, HT patients had slightly worse lipid profile (baseline low-density lipoprotein 98.2 ± 37.0 vs. 94.8 ± 38.3 mg/dl, *P* < 0.001), and as expected had higher baseline TSH value (mean 4.6 vs. 1.8 mIU/L, *P* < 0.001) (Tables 1, 2).

**TABLE 1:** Baseline patients characteristics.

	Patients with hypothyroidism, *N* = 1,922	Control patients, *N* = 23,673	*P*-value
Age, y, (mean ± SD)	70.3 ± 10.4	66.0 ± 11.8	< 0.001
Gender, Female (%)	34.2	26.1	< 0.001
**Medical History of:**			
Diabetes Mellitus, (%)	50.2	48.2	0.092
Hypertension, (%)	76.9	76.5	0.240
Smoking, (%)	22.4	27.6	0.133
COPD, (%)	11.5	8.6	0.120
PVD, (%)	4.7	3.5	0.118
AF, (%)	10.8	7.7	< 0.001
Anemia (%)*	54.1	50.8	0.052
CKD (eGFR < 60ml/min), (%)	25.1	18.7	0.040
Prior Stroke, (%)	8.8	6.0	0.210
Prior CHF, (%)	12.5	11.7	0.542
Prior CABG, (%)	14.8	13.5	0.208
Prior Malignancy, (%)	12.1	10.0	0.490
Dementia, (%)	2.9	1.8	0.004
**Drugs**			
Aspirin, (%)	91.7	95.9	0.242
OAC, (%)	14.9	7.1	< 0.001
ACE I/ARB, (%)	78.2	82.7	0.144
BB, (%)	76.9	79.8	0.448
Statins, (%)	94.1	96.4	0.383
Diuretics, (%)	30.4	25.7	0.210
VKA, (%)	9.3	4.5	< 0.001
NOAC, (%)	5.6	2.7	< 0.001
Insulin, (%)	15.6	12.7	< 0.001
Clopidogrel, (%)	83.8	76.3	0.272
Ticagrelor, (%)	10.5	13.3	0.102
Prasugrel, (%)	7.0	9.0	0.218
Levothyroxine, (%)	70.2	0.0	–
ARNI, (%)	0.3	0.2	0.201
SGLT2 I, (%)	1.5	2.4	0.020

SD, standard deviation; COPD, chronic obstructive pulmonary disease; PVD, peripheral vascular disease; AF, atrial fibrillation; CKD, chronic kidney disease; CHF, congestive heart failure; CABG, coronary artery bypass graft surgery; EF, ejection fraction; eGFR, estimated glomerular filtration rate; OAC, oral anticoagulants; ACE I/ARB, angiotensin converting enzyme inhibitors/angiotensin-receptor blockers; VKA, vitamin K antagonist; NOAC, novel oral anticoagulants; ANRI, angiotensin receptor-neprilysin inhibitor; SGLT2 I, sodium-glucose cotransporter-2 inhibitors; SD, standard deviation.

*Hb < 14 gr/dl for men and < 12gr/dl for women.

**TABLE 2:** Main clinical parameters at presentation.

	Patients with hypothyroidism, *N* = 1,922	Control patients, *N* = 23,673	*P*-value
Estimated EF, % (mean ± SD)	54.1 ± 9.7	54.3 ± 9.2	0.120
Radial access (%)	67.8	70.4	0.342
PCI for ACS, (%)	54.5	52.3	0.569
Disease vessel number, (mean ± SD)	2.2 ± 0.8	2.2 ± 0.8	0.352
CTO PCI, (%)	9.9	11.0	0.180
Bifurcation lesion, (%)	12.4	12.9	0.322
Severe calcification, (%)	19.0	12.0	< 0.001
Severe state (shock), (%)	1.3	1.2	0.443
Hemoglobin, gr/dl (mean ± SD)	12.6 ± 1.7	13.3 ± 1.8	0.148
Platelets, K/ul (mean ± SD)	231.4 ± 80.0	231.1 ± 74.1	0.411
Creatinine, mg/dl (median [IQR])	1.2 (0.7, 1.5)	1.1 (0.7, 1.4)	0.220
eGFR, ml/min/1.73 m^2^ (mean ± SD)	78.4 ± 28.6	82.4 ± 28.1	0.410
Total cholesterol, mg/dl (mean ± SD)	172.1 ± 45.4	167.7 ± 45.4	0.024
Triglycerides, mg/dl (mean ± SD)	157.9 ± 101.0	156.2 ± 110.5	0.110
WBC, K/ul (mean ± SD)	8.3 ± 4.0	8.7 ± 5.8	0.181
LDL, mg/dl (mean ± SD)	98.2 ± 37.0	94.8 ± 38.3	< 0.001
TSH, mIU/L (median [IQR])*	4.6 (2.2, 8.1)	1.8 (1.1, 2.8)	< 0.001

SD, standard deviation; CTO, chronic total occlusion; WBC, white blood cells; LDL, low-density lipoprotein; TSH, thyroid stimulating hormone; IQR, interquartile range.

*Due to abnormal distribution of TSH values, IQR is presented.

Regarding patient presentation, both groups presented with preserved mean left ventricular ejection fraction (∼54%), while the minority of patients, about 1%, presented with hemodynamic instability. PCI in an urgent ACS setting was not significantly different between the groups and included 52–54% of cases (*P* = 0.569). In terms of baseline medications, HT patients were more frequently treated with vitamin K antagonist (9.3% vs. 4.5%, *P* < 0.001), novel oral anticoagulants (5.6% vs. 2.7%, *P* < 0.001), and insulin (15.6% vs. 12.7%, *P* < 0.001). A trend toward less frequent use of potent P2Y12 inhibitors was noticed in this group. Additionally, coronary angiography differed between groups in that severe calcification was more frequent in HT patients (19.0% vs. 12.0%, *P* < 0.001) (Table 2).

**FIGURE 3:**
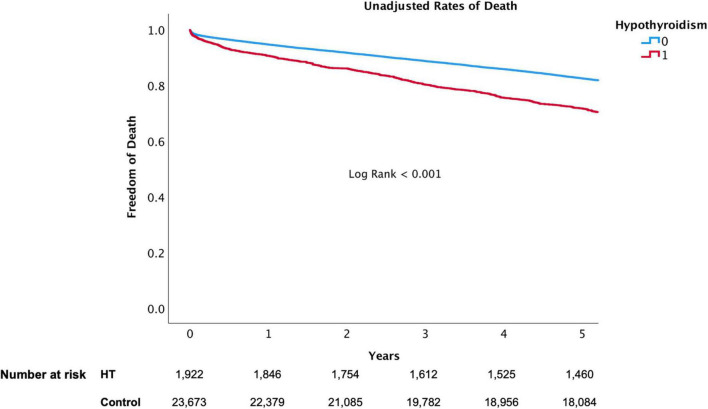
5-Year all-cause mortality of hypothyroidism versus euthyroid patients.

**FIGURE 4:**
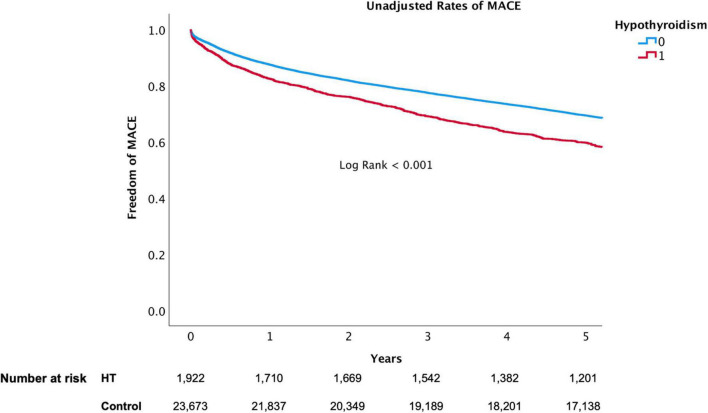
5-Year MACE of hypothyroidism versus euthyroid patients.

**FIGURE 5:**
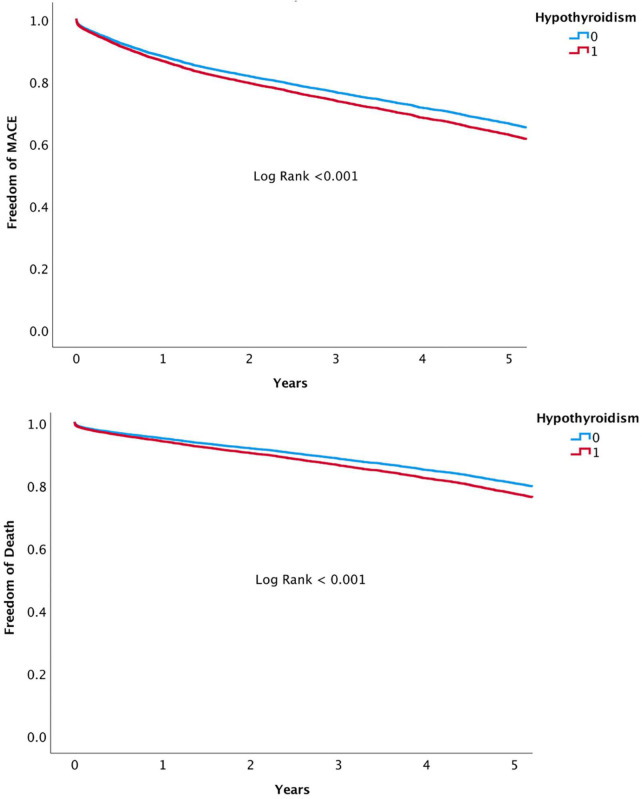
Adjusted rates of all-cause mortality and MACE at 5 years.

The presence of HT was associated with a significant higher risk of mortality and MACE over 5-year follow-up (Figures 3, 4, respectively). At 1-year, mortality and MACE were 8.4% vs. 5.8% and 18.8% vs. 12.7%, respectively (*P* < 0.001). At 5-year, mortality and MACE were 26.9% vs. 20.3% and 40.3% vs. 29.4%, respectively (*P* < 0.001). Following multivariate analysis, using Cox proportional hazards regression and increased TSH as a continuous parameter, the presence of increased TSH was still associated with significant higher rates of mortality (HR, 1.06; CI, 1.02–1.11; *P* < 0.001) and MACE (HR, 1.07; CI, 1.02–1.12; *P* < 0.001), (Table 3 and Figure 5). A propensity match score was able to form 672 matched pairs of HT and control patients, showing similar results; Following Cox regression, patients presenting with HT demonstrated higher rates 5-year mortality and MACE than patients without HT (HR 1.19; CI: 1.06–1.74 and HR 1.18; CI: 1.09–1.48, respectively; *p* < 0.001).

**TABLE 3:** Cox regression analyses for mortality and MACE at 5 years.

Parameter	Mortality		MACE	
	HR (95% CI)	*P*-value	HR (95% CI)	*P*-value
Age (per year)	1.02 (1.01–1.02)	< 0.001	1.01 (1.01–1.02)	< 0.001
Gender, Female	0.98 (0.92–1.05)	0.421	0.99 (0.94–1.04)	0.790
CKD	1.47 (1.36–1.58)	< 0.001	1.43 (1.34–1.52)	< 0.001
DM	1.15 (1.07–1.24)	< 0.001	1.17 (1.12–1.20)	< 0.001
HTN	0.84 (0.71–1.01)	0.148	0.97 (0.91–1.04)	0.347
Prior PVD	1.46 (1.32–1.57)	< 0.001	1.44 (1.3–1.59)	< 0.001
Prior AF	0.72 (0.69–1.01)	0.102	0.84 (0.76–0.93)	< 0.001
Anemia	1.03 (0.95–1.12)	0.662	1.01 (0.95–1.07)	0.820
Prior dementia	1.32 (1.01–1.60)	< 0.001	1.30 (1.11–1.50)	< 0.001
Acute MI	1.30 (1.18–1.43)	< 0.001	1.31 (1.20–1.44)	< 0.001
Heavy calcification	1.34 (1.23–1.44)	0.080	1.36 (1.2–1.46)	< 0.001
EF (per 1%)	0.98 (0.97–0.99)	< 0.001	0.99 (0.97–0.99)	< 0.001
TSH (per 1 mIU/L)	1.06 (1.02–1.11)	< 0.001	1.07 (1.02–1.12)	< 0.001

MACE, major adverse cardiac event; HR, hazard ratio; CKD, chronic kidney disease; DM, diabetes mellitus; CHF, congestive heart failure; PVD, peripheral vascular disease; AF, Atrial fibrillation; MI, myocardial infarction; EF, ejection fraction; TSH, thyroid stimulating hormone.

## Discussion

Our study assessed the impact of HT on the outcomes of patients undergoing PCI and found an increased risk of both death and MACE following PCI among HT patients compared to counterpart patients. To the best of our knowledge, it is the largest outcome analysis of HT patients following PCI. This confirms prior smaller retrospective studies, which demonstrated a significant association of HT with all-cause mortality (10, 17–19).

The previous retrospective study by Zhang et al., based on the Mayo Clinic PCI registry, examined a similar research question (12). This study showed that HT was associated with a higher incidence of MACCE (MACE plus cerebral events) compared with euthyroid patients undergoing PCI. Moreover, it showed that adequate hormone replacement therapy with TSH corrected to the normal range was associated with a lower incidence of MACCE compared to untreated or inadequately treated patients (12). Related to our study, although the initial cohort of this study was quite similar and stands at about 25,000 patients, due to missing data, and strict exclusion criteria, only 686 patients (<3%) were included in the hypothyroid group of this previous study. It implies a possible underestimation of this patients population and a potential for selection bias in this study. In addition, the median duration of follow-up in this study was shorter (3 vs. 5 years in our study). Furthermore, the long (e.g., 7-year) time interval passed since this study allow us to evaluate the changes in PCI performance (such as advanced-generation stents and potent P2Y12 inhibitors) in the context of patients with HT.

As for the etiology of the increased mortality and MACE incidence in HT patients, our study confirms previous observations that patients with HT have a significantly higher prevalence of comorbidities associated with a high cardiovascular risk profile, such as prior myocardial infarction, diabetes mellitus, hypertension, COPD, and chronic renal disease. However, using propensity score matching, we were also able to demonstrate an independent effect of HT on adverse clinical outcomes. This observation supports previous studies that demonstrated many pathophysiological effects of thyroid hormones on the cardiovascular systems, and make it plausible that HT itself is not only a risk marker but rather a risk target that should be treated to decrease mortality and cardiovascular morbidities (7, 20). Previously demonstrated effects that linked HT and the cardiovascular system include changes of arterial compliance, diastolic blood pressure, endothelial dysfunction, hyperlipidemia and intima-media thickening, which is a known surrogate marker for atherosclerosis (8, 11). While not completely understood, it is apparent that HT affects many physiological processes including systemic diseases with known detrimental prognostic cardiovascular aspects, and some effect the cardiovascular system directly.

Subclinical hypothyroidism correlates with worse cardiovascular outcome to a lesser extent than HT. To date, there is no recommendation to prescribe thyroid hormone replacement therapy to SHT patients (21, 22). Due to seemingly SHT underdiagnosis in our study, we chose to exclude SHT. We, however, performed Cox proportional hazards regression with increased TSH as a continuous parameter, including mildly elvated TSH values due to suboptimal treatment in HT patients. The significant higher rates of mortality with increased TSH support previous observation regarding worse cardiovascular outcome in HT patients with suggested worst prognosis in untreated patients. A recently published large retrospective cohort study examined 705,307 adults who received thyroid hormone treatment from the US Veterans Health Administration Corporate Data Warehouse and demonstrated a highed cardiovascular mortality among patients with TSH and free thyroxine levels that were not controlled within the normal range, whether it resulted in HT or exogenous hyperthyroidism (23). Our study demonstrates for the first time that a cohort of patients with HT suffer higher mortality and MACE rates following PCI. Our Propensity score matching analysis strengthen this observation as HT was independently associated with significant higher rates of mortality. Moreover, TSH as a continuous parameter in a Cox proportional hazards regression was still associated with significant higher rates of mortality. Whether it implies a possible beneficial effect of TSH targeted therapy on mortality remains to be explored. Further studies, preferably randomized prospective studies, are needed to examine the clinical benefit of thyroid hormone replacement therapy targeted to a middle-normal TSH level in patients undergoing PCI.

Our study has several limitations. First, it is based on an all-comer PCI registry from two medical centers, and thus is liable to the limitations of observational study design. Second, the definition of HT was based on medical records and TSH values that were not available for all patients. We also lacked free T4 levels, data regarding TPO/Tg antibodies and the etiology of HT. Finally, many patients (∼8.5% of the cohort) were excluded from the final analysis due to missing data. Nevertheless, to date, this is the largest contemporary study examining the independent impact of HT on outcomes of patients undergoing PCI, showing significantly worse outcomes.

In conclusion, our findings strengthen previous retrospective studies demonstrating the association between HT and worse outcomes in coronary artery disease undergoing PCI. As mortality and MACE were significantly higher for patients with HT, it may suggest TSH targeted therapy in this patients population. As our study design was not targeted to answer this question, future interventional studies are warranted to establish methods to mitigate this risk.

## Data availability statement

The raw data supporting the conclusions of this article will be made available by the authors, without undue reservation.

## Ethics statement

The studies involving human participants were reviewed and approved by Rabin Medical Center Ethics Committee. Written informed consent for participation was not required for this study in accordance with the national legislation and the institutional requirements.

## Author contributions

BC and LiP: study concept and design, methodology, interpretation of data, and drafting of the manuscript. TB: database queries formation. LeP: statistical analysis. LiP, TB, HV, PC, KO, YT, AL, and RK: establishing and maintaining the PCI database. All authors contributed to the data and final manuscript were critically discussed, revised, and approved.
